# Effects of extracellular vesicles derived from oral bacteria on osteoclast differentiation and activation

**DOI:** 10.1038/s41598-022-18412-4

**Published:** 2022-08-20

**Authors:** Hyun Young Kim, Min-Kyoung Song, Younggap Lim, Ji Sun Jang, Sun-Jin An, Hong-Hee Kim, Bong-Kyu Choi

**Affiliations:** 1 Department of Oral Microbiology and Immunology, School of Dentistry, Seoul National University, 101 Daehak-ro, Jongno-gu, Seoul, 03080 Republic of Korea; 2 Department of Internal Medicine, Seoul National University Hospital, Seoul, Republic of Korea; 3 Department of Cell and Developmental Biology, School of Dentistry, Seoul National University, Seoul, Republic of Korea; 4 Dental Research Institute, School of Dentistry, Seoul National University, Seoul, Republic of Korea

**Keywords:** Pathogens, Osteoimmunology, Pattern recognition receptors

## Abstract

Dysbiosis of the oral microbiota plays an important role in the progression of periodontitis, which is characterized by chronic inflammation and alveolar bone loss, and associated with systemic diseases. Bacterial extracellular vesicles (EVs) contain various bioactive molecules and show diverse effects on host environments depending on the bacterial species. Recently, we reported that EVs derived from *Filifactor alocis,* a Gram-positive periodontal pathogen, had osteoclastogenic activity. In the present study, we analysed the osteoclastogenic potency and immunostimulatory activity of EVs derived from the Gram-negative periodontal pathogens *Porphyromonas gingivalis* and *Tannerella forsythia*, the oral commensal bacterium *Streptococcus oralis*, and the gut probiotic strain *Lactobacillus reuteri*. Bacterial EVs were purified by density gradient ultracentrifugation using OptiPrep (iodixanol) reagent. EVs from *P. gingivalis*, *T. forsythia*, and *S. oralis* increased osteoclast differentiation and osteoclstogenic cytokine expression in osteoclast precursors, whereas EVs from *L. reuteri* did not. EVs from *P. gingivalis*, *T. forsythia*, and *S. oralis* preferentially activated Toll-like receptor 2 (TLR2) rather than TLR4 or TLR9, and induced osteoclastogenesis mainly through TLR2. The osteoclastogenic effects of EVs from *P. gingivalis* and *T. forsythia* were reduced by both lipoprotein lipase and polymyxin B, an inhibitor of lipopolysaccharide (LPS), while the osteoclastogenic effects of EVs from *S. oralis* were reduced by lipoprotein lipase alone. These results demonstrate that EVs from periodontal pathogens and oral commensal have osteoclastogenic activity through TLR2 activation by lipoproteins and/or LPS.

## Introduction

Periodontitis is a chronic inflammatory disease initiated by polymicrobial infection of oral bacterial species, leading to tissue inflammation, alveolar bone loss, and diverse systemic diseases^1^. Oral commensal bacteria have immunomodulatory activities and play an important role in host-microbe homeostasis. However, the predominance of Gram-negative periodontal pathogens such as ‘red complex’ bacteria (*Porphyromonas gingivalis*, *Tannerella forsythia*, and *Treponema denticola*) is known as a major cause of periodontitis^2^. Recently, *Filifactor alocis*, a Gram-positive bacterium, has also been regarded as an emerging periodontal pathogen that is frequently detected in periodontal pockets in periodontitis patients and considered a co-occurrence group with periodontal pathogens, including *P. gingivalis* and *T. forsythia*^3^. Periodontal pathogens have various virulence factors, including microbe-associated molecular patterns (MAMPs), proteases, toxins, and complement inhibitors, that play an important role in the progression of periodontitis^4^. However, probiotic strains exhibit beneficial effects on the treatment and prevention of periodontal diseases^5^.

Osteoclasts are multinucleated cells that originate from bone marrow-derived haematopoietic precursor cells and play an important role in the regulation of bone homeostasis by resorbing bone^6^. Excessive activation of osteoclasts causes an imbalance in bone metabolism, leading to the progression of bone loss. The differentiation and activation of osteoclasts are mainly regulated by receptor activator of nuclear factor-κB ligand (RANKL), macrophage colony-stimulating factor (M-CSF), and proinflammatory cytokines such as TNF-α, IL-6 and IL-1^7^. Osteoclasts express Toll-like receptors (TLRs), which are essential pattern recognition receptors for innate immunity against microbial infections that recognize MAMPs^8^. Bacterial-derived MAMPs, such as lipopolysaccharide (LPS), lipoproteins, flagellin, DNA and RNA, can modulate osteoclast differentiation and activation through TLR signalling pathways^9^.

Bacterial extracellular vesicles (EVs) are bacteria-derived nanosized vesicles surrounded by lipid bilayers and have diverse effects on host environments. They carry various bioactive molecules including MAMPs, proteins, lipids, sugars and nucleic acids, of their originated bacteria and transfer the molecules to host cells^10^. The immunostimulatory activities of bacterial EVs are dependent on the profiles of their molecular components. EVs from Gram-negative bacteria have LPS, which is a representative TLR4 ligand and regarded as a major MAMP for inducing proinflammatory responses in host cells^11^. In EVs from Gram-positive bacteria, bacterial lipoproteins are known as major TLR2 ligands^12^. EVs from probiotic strains harbour immunomodulatory molecules that have beneficial properties, such as a reduction in proinflammatory cytokines and an increase in epithelial barrier functions^13,14^.

Recently, we reported for the first time that EVs derived from the Gram-positive periodontal pathogen *F. alocis* induced osteoclast differentiation through TLR2^15^. In the present study, we evaluated the effects of EVs from Gram-negative periodontal pathogens *P. gingivalis* and *T. forsythia*, oral commensal bacterium *Streptococcus oralis*, and a gut probiotic strain *Lactobacillus reuteri* on osteoclast differentiation.

## Results

### Isolation and characterization of bacterial EVs

**Figure 1:**
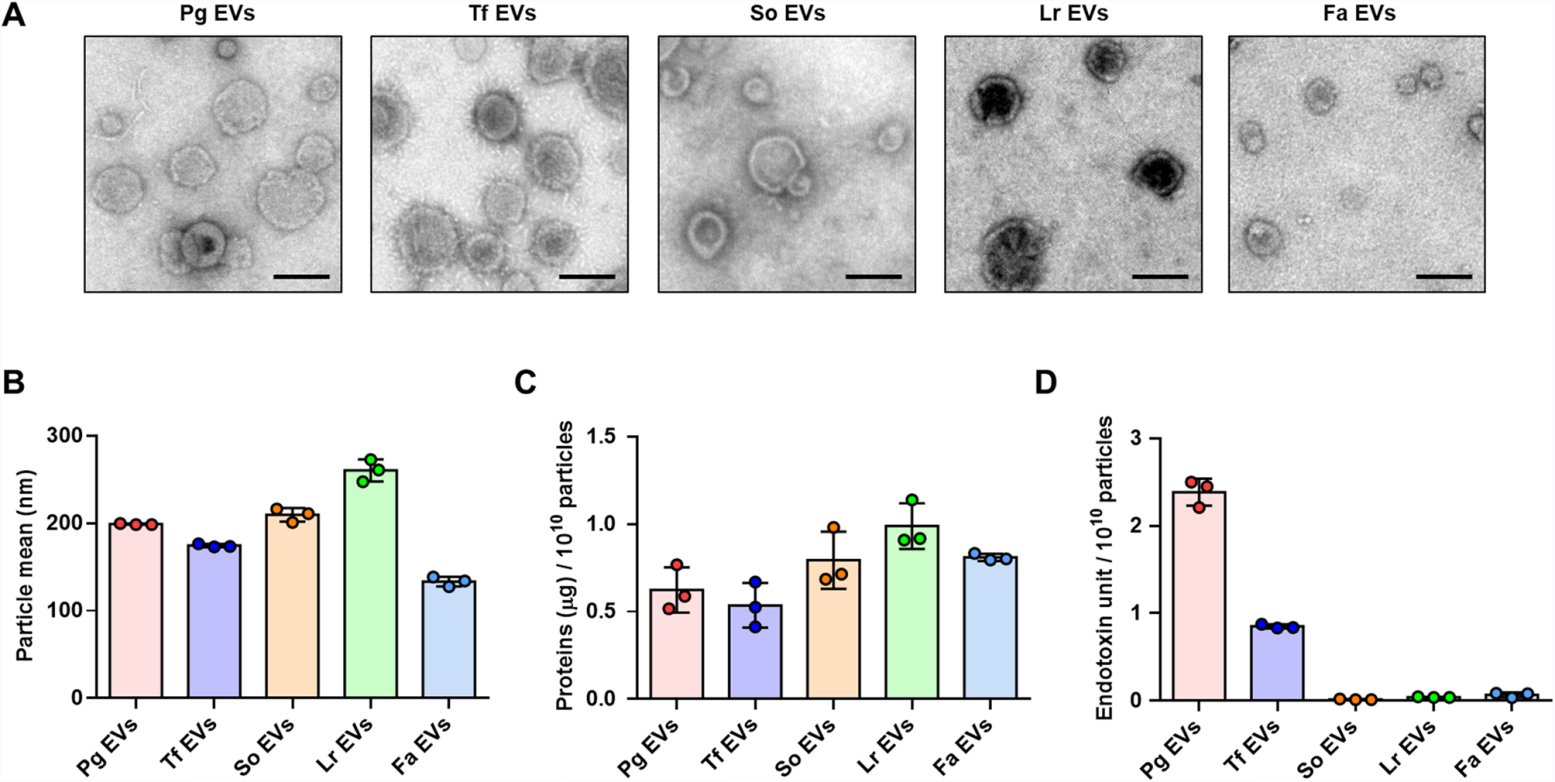
Characterization of bacterial EVs. (**A**) TEM photographs showing morphology of EVs released from *P. gingivalis* (Pg EVs), *T. forsythia* (Tf EVs), *S. oralis* (So EVs), *L. reuteri* (Lr EVs), and *F. alocis* (Fa EVs) (scale bar: 100 nm). (**B**) The mean size (nm) of EVs was analysed by NTA. (**C**) Proteins (μg)/10^10^ particles of EVs. (**D**) Endotoxin unit/10^10^ particles of EVs was measured by the LAL assay. Particle numbers and total protein amount (μg) were measured by NTA and BCA assay, respectively. The graphs show the mean values ± standard deviations of three biological replicates.

EVs from periodontal pathogens (*P. gingivalis*, *T. forsythia*, and *F. alocis*), an oral commensal strain (*S. oralis*), and a gut probiotic strain (*L. reuteri*) were purified by buoyant density gradient ultracentrifugation. The morphology of bacterial EVs were examined by transmission electron microscopy (TEM), and the mean size and particle number of EVs were determined by nanoparticle tracking analysis (NTA). The TEM image showed that EVs released from *P. gingivalis* (Pg EVs), *T. forsythia* (Tf EVs), *S. oralis* (So EVs), *L. reuteri* (Lr EVs), and *F. alocis* (Fa EVs) were nanosized and vesicular structures without residual bacterial cells (Fig. 1A). NTA showed that the mean size was 199.1 nm for Pg EVs, 174.5 nm for Tf EVs, 209.5 nm for So EVs, 260.5 nm for Lr EVs, and 133.4 nm for Fa EVs (Fig. 1B). Because NTA measures the same particle larger than TEM^16^ the mean size of bacterial EVs by NTA appears to be larger than that of TEM image. The EV proteins (μg)/10^10^ EV particles was 0.62 for Pg EVs, 0.54 for Tf EVs, 0.79 for So EVs, 0.99 for Lr EVs, and 0.81 for Fa EVs (Fig. 1C). Next, to determine the endotoxin unit (EU) of EVs from Gram-negative periodontal pathogens (Pg EVs and Tf EVs), the LAL test was conducted. As shown in Fig. 1D, endotoxins unit/10^10^ EV particles were detected both in Pg EVs and Tf EVs, and the endotoxins unit/10^10^ EV particles was 2.39 for Pg EVs and 0.85 for Tf EVs. EVs from Gram-positive bacteria (So EVs, Lr EVs, and Fa EVs) were also examined to evaluate endotoxin contamination. No endotoxin activity was detected in EVs from Gram-positive bacteria (So EVs, Lr EVs, and Fa EVs). These results suggested that we obtained structurally intact and pure bacterial EVs.

### Immunostimulatory effects of oral bacterial EVs on osteoclast precursors

**Figure 2:**
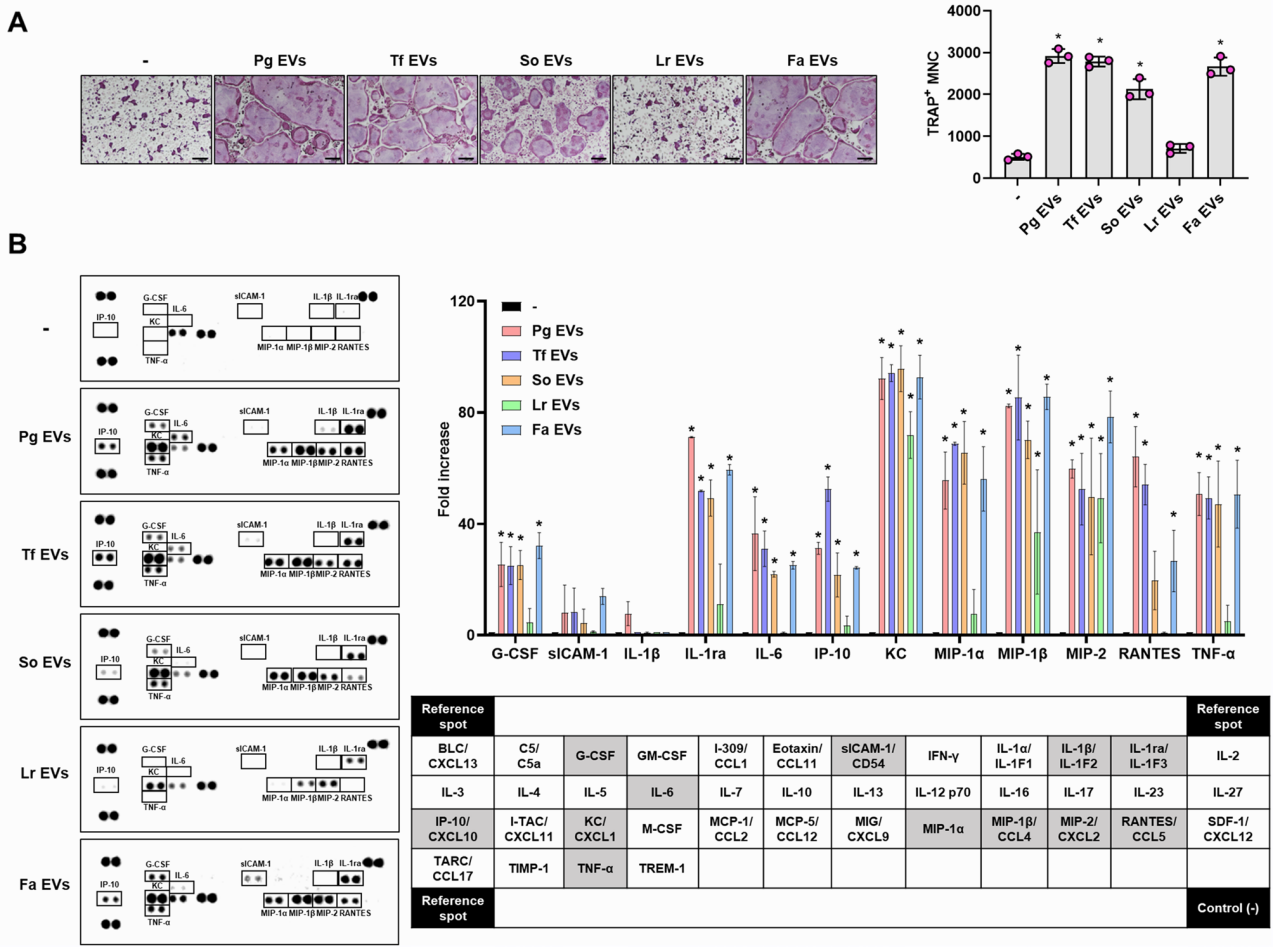
Effects of bacterial EVs on osteoclast differentiation and activation. (**A**) Osteoclast precursors were stimulated with bacterial EVs (10 μg/ml EV protein) in the presence of M-CSF (30 ng/ml) for 3 days. The mature osteoclasts were stained with TRAP, and representative images from triplicate samples are shown (left panel, scale bar: 200 μm). The number of TRAP^+^ mature osteoclasts was counted (right panel). The experiments were performed at least three times independently. The graphs show the mean values ± standard deviations of a representative experiment. (**B**) Osteoclast precursors were stimulated with bacterial EVs (10 μg/ml EV protein) in the presence of M-CSF (30 ng/ml) for 24 h. Then, cytokines in the supernatants were analysed using a Cytokine Array Kit. Dot intensity was measured by densitometry using Bio-Rad Image Lab software. The fold increase (right upper panel) of the treatment groups was determined by dividing the dot intensity value by that of nontreatment group. The graphs show the mean values ± standard deviations of two biological replicates. Statistical significance was determined by one-way ANOVA (**A**) or by two-way ANOVA (**B**). *P < 0.05 compared with the nontreatment group ( −).

Osteoclasts are multinucleated giant cells that originated from haematopoietic lineage mononuclear cells. Local infection of periodontal pathogens can cause osteoclast differentiation and bone resorption^17^. Recently, we determined that EVs from the Gram-positive periodontal pathogen *F. alocis* efficiently induce osteoclast differentiation and activation^15^. We used Fa EVs as a positive control to induce osteoclast differentiation in further experiments in this study. To evaluate the osteoclastogenic potencies of EVs from Gram-negative periodontal pathogens (Pg EVs and Tf EVs), an oral commensal strain (So EVs), and a gut probiotic strain (Lr EVs), committed osteoclast precursors were stimulated with bacterial EVs (10 μg/ml) without RANKL. As shown in Fig. 2A, EVs from Pg EVs and Tf EVs remarkably induced osteoclast differentiation, similar to Fa EVs. Interestingly, So EVs also induced osteoclast differentiation. However, Lr EVs did not induce differentiation to mature osteoclasts. The osteoclastogenic potencies of EVs from mid-log phase were similar to those of EVs from 48 h culture supernatants (Supplementary Fig. S1). Next, to identify the immunostimulatory effects of bacterial EVs on osteoclast precursors, we stimulated osteoclast precursors with each bacterial EV and analysed 40 cytokines in the culture supernatants using a cytokine array (Fig. 2B). Bacterial EVs, which have osteoclastogenic potencies (Pg EVs, Tf EVs, So EVs, and Fa EVs), increased the expression of G-CSF, IL-1ra, IL-6, IP-10, KC, MIP-1α, MIP-1β, MIP-2, RANTES and TNF-α in osteoclast precursors. Lr EVs increased the expression of KC, MIP-1β, and MIP-2, but did not induce major osteoclastogenic cytokines, such as TNF-α, IL-6, and IL-1β. These results indicate that EVs from oral bacteria including periodontal pathogens but not probiotic strain efficiently induce osteoclast differentiation and activation.

### EVs from oral bacteria induce osteoclastogenesis preferentially via TLR2

**Figure 3:**
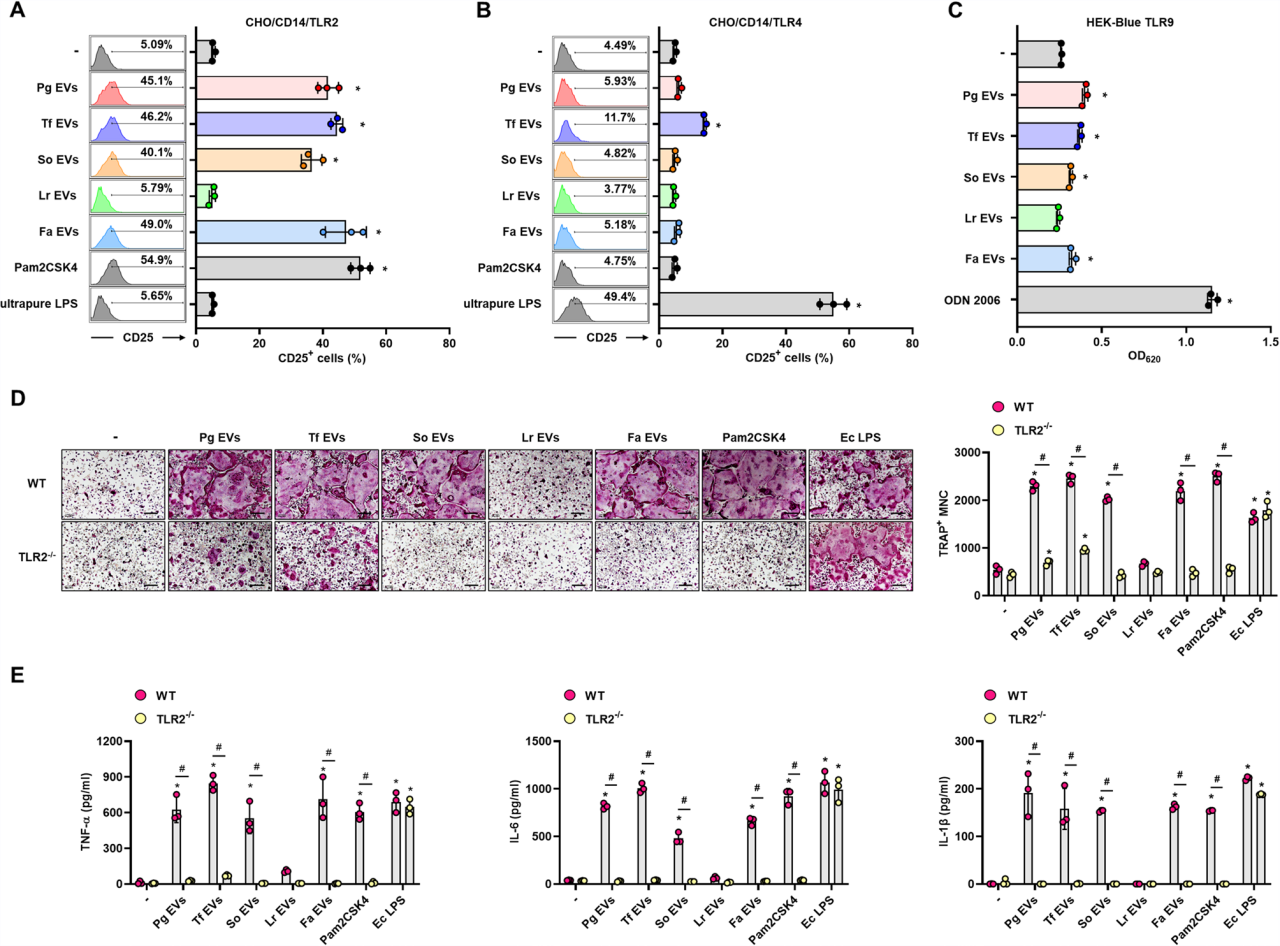
TLR2 activation by bacterial EVs in osteoclast precursors. (**A**, **B**) CHO/CD14/TLR2 or CHO/CD14/TLR4 cells were stimulated with bacterial EVs (1 μg/ml EV protein) for 20 h. CD25 expression by TLR activation was measured by flow cytometry. Pam2CSK4 (100 ng/ml) and ultrapure *E. coli* LPS (100 ng/ml) were used as TLR2 and TLR4 ligands, respectively. (**C**) HEK-Blue TLR9 cells were stimulated with bacterial EVs (1 μg/ml EV protein) for 24 h. SEAP expression by TLR9 activation was measured by a spectrophotometer at 620 nm. ODN 2006 (1 μg/ml) was used as a TLR9 ligand. (**D**) WT and TLR2^−/−^ osteoclast precursors were stimulated with bacterial EVs (10 μg/ml EV protein) in the presence of M-CSF (30 ng/ml) for 3 days. The number of TRAP^+^ mature osteoclasts was counted (right panel). The experiments were performed at least three times independently. The graphs show the mean values ± standard deviations of a representative experiment. (**E**) WT and TLR2^−/−^ osteoclast precursors were stimulated with bacterial EVs (10 μg/ml EV protein) in the presence of M-CSF (30 ng/ml) for 24 h. Osteoclastogenic cytokines (TNF-α, IL-6, and IL-1β) in the culture supernatants were measured by ELISA. Pam2CSK4 (1 μg/ml) and *E. coli* LPS (1 μg/ml) were used as positive controls. The experiments were performed at least three times independently. The graphs show the mean values ± standard deviations of a representative experiment. Statistical significance was determined by one-way ANOVA (**A**, **B**, and **C**) or by two-way ANOVA (**D**, **E**). *P < 0.05 compared with the nontreatment group ( −). ^#^*P* < 0.05 compared with the indicated group.

Oral bacteria can activate TLRs in osteoclast precursors to regulate osteoclast differentiation and activation^18^. To determine the TLR2-, TLR4-, or TLR9-activating abilities of bacterial EVs, we used the reporter cell lines CHO/CD14/TLR2, CHO/CD14/TLR4, and HEK-Blue TLR9, which highly express human TLR2, TLR4, and TLR9, respectively. We measured the TLR-activating ability of bacterial EVs at a low dose (1 μg/ml) because Pg EV-stimulated CHO/CD14 cells at 10 μg/ml were not stained with FITC-conjugated anti-CD25 antibody, which may be due to the degradation of cell surface molecules by the high proteolytic activity of Pg EVs (data not shown). As shown in Fig. 3A and B, Pg EVs, Tf EVs, So EVs, and Fa EVs highly activated TLR2. Interestingly, Pg EVs and TF EVs, EVs of Gram-negative periodontal pathogens, preferentially activated TLR2. Although Tf EVs activated TLR4, the activation potential was much less than that of TLR2. Pg EVs, Tf EVs, So EVs, and Fa EVs slightly activated TLR9 (Fig. 3C). Lr EVs did not activate TLR2, TLR4 or TLR9 in our experimental conditions. To identify the role of TLR2 in bacterial EV-induced osteoclast differentiation, WT or TLR2^−/−^ osteoclast precursors were stimulated with bacterial EVs without RANKL. Pg EVs and Tf EVs slightly induced osteoclast differentiation in TLR2^−/−^ osteoclast precursors, but So EVs and Fa EVs did not induce it at all (Fig. 3D). Lr EVs did not induce osteoclast differentiation in either WT or TLR2^−/−^ osteoclast precursors. Likewise, ELISA data showed that major osteoclastogenic cytokines (TNF-α, IL-6 and IL-1β) were increased in WT osteoclast precursors by Pg EVs, Tf EVs, So EVs, and Fa EVs (Fig. 3E). However, in TLR2^−/−^ osteoclast precursors, osteoclastogenic cytokines were not induced by all EVs. These results suggest that TLR2 may be the major immune receptor for the recognition of oral bacterial EVs, leading to osteoclast differentiation and activation.

### Effects of lipoproteins and LPS from oral bacterial EVs on osteoclast differentiation

**Figure 4:**
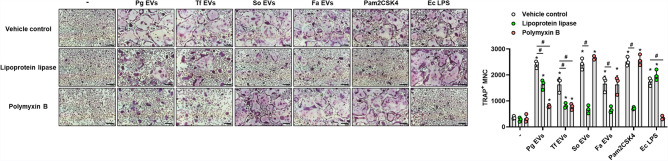
Role of lipoproteins and LPS in oral bacterial EVs in osteoclast differentiation. Oral bacterial EVs were incubated with 50 μg/ml lipoprotein lipase or polymyxin B at 37°C for 16 h. Then, osteoclast precursors were stimulated with oral bacterial EVs (10 μg/ml EV protein) for 3 days. For the negative control, lipoprotein lipase or polymyxin B without bacterial EVs was administered to the nontreatment group ( −). The number of TRAP^+^ mature osteoclasts was counted (right panel). The experiments were performed at least three times independently. Pam2CSK4 (1 μg/ml) and *E. coli* LPS (1 μg/ml) were used as positive controls. The graphs show the mean values ± standard deviations of a representative experiment. Statistical significance was determined by two-way ANOVA. *P < 0.05 compared with the nontreatment group ( −). ^#^*P* < 0.05 compared with the indicated group.

Bacterial lipoproteins and LPS are known as major TLR2 and TLR4 ligands of bacterial EVs, respectively^11,12^. Recently, we identified that lipoproteins of Fa EVs are major inducers of osteoclast differentiation^15^. To identify the role of lipoproteins and LPS in oral bacterial EVs, we incubated the EVs with lipoprotein lipase or polymyxin B and collected the EVs by ultracentrifugation to remove residual treatments. As shown in Fig. 4, both lipoprotein lipase and polymyxin B reduced the osteoclastogenic potency of Pg EVs and Tf EVs, while the osteoclastogenic potency of So EVs and Fa EVs was reduced by lipoprotein lipase alone. These results indicate that both bacterial lipoproteins and LPS of EVs from Gram-negative periodontal pathogens may act as TLR2 ligands leading to osteoclast differentiation, while lipoproteins are major TLR2 ligands of EVs from Gram-positive oral bacteria for osteoclastogenesis.

## Discussion

Bacteria naturally release EVs that affect host physiology without direct interaction between bacteria and host cells. EVs released from pathogens exhibit pathogenic properties, while EVs released from probiotics seem to be beneficial. In this study, we first compared the osteoclastogenic potency of EVs derived from periodontal pathogens, an oral commensal, and a probiotic strain. EVs from periodontal pathogens (Pg EVs, Tf EVs, and Fa EVs) and an oral commensal strain (So EVs) induced the expression of osteoclastogenic cytokines and osteoclast differentiation but not the probiotic strain (Lr EVs). We also demonstrated that oral bacterial EVs mainly activated TLR2 rather than TLR4 or TLR9.

Recently, we reported that EVs from Gram-positive periodontal pathogen (Fa EVs) induced osteoclast differentiation and bone resorption through TLR2^15^. In the present study, we observed that EVs derived from the Gram-negative periodontal pathogens *P. gingivalis* and *T. forsythia* also preferentially activate TLR2 rather than TLR4 and induce osteoclastogenesis mainly via TLR2. TLR2 signalling by *P. gingivalis* and *T. forsythia* has been reported. *P. gingivalis* increased TNF-α production in macrophages through TLR2 signalling, leading to alveolar bone loss^19^. TLR2 signalling plays an important role in *T. forsythia*-mediated alveolar bone loss and activation of osteoclasts^20^. The osteoclastogenic activity of Pg EVs and Tf EVs was reduced by lipoprotein lipase treatment, suggesting that lipoproteins are active molecules, as shown in our previous study^15^. Jain et al. reported that *P. gingivalis* induced inflammation via TLR2 signalling, which was attenuated by lipoprotein lipase treatment^21^. The attenuated osteoclastogenic activity of Pg EVs and Tf EVs in TLR2^−/−^ osteoclast precursors supports the role of lipoproteins as TLR2 ligands. As with lipoprotein lipase treatment, the LPS inhibitor polymyxin B also reduced the osteoclastogenic activity of Pg EVs and Tf EVs, indicating that LPS was also involved. Although *P. gingivalis* LPS has heterogeneous lipid A structures that can activate both TLR4 and TLR2^22^, TLR2 may be more important than TLR4 in that *P. gingivalis* LPS induced alveolar bone loss through TLR2 in mice^23^. Polymyxin B is known to bind to LPS, thus inhibiting the interaction of LPS with TLRs. Recently, it has been reported that EVs from *Acinetobacter baumannii*, a Gram-negative pathogen, directly bind to polymyxin B, leading to the protection of *A. baumannii* cells from lysis by polymyxin B^24^. With regard to this finding, it can be speculated that polymyxin B can bind to LPS on EVs of *P. gingivalis* and *T. forsythia*, thus preventing the interaction of LPS with TLRs. TLR signalling can affect progression of not only osteoporosis but also several systemic diseases including Alzheimer's disease, atherosclerosis, and diabetes, which are closely related to periodontitis. In periodontitis patients, EVs can be released from periodontal pathogens in the local subgingival sites and spread freely to systemic sites leading to disease progression. Considering these points, periodontal pathogen EVs and their TLR ligands might affect progression and exacerbation of periodontitis-related systemic inflammatory diseases.

EVs from the oral commensal *S. oralis* also showed high TLR2 activation ability and osteoclastogenic potency as EVs of periodontal pathogens. Oral commensal bacteria may have negative effects on host bone metabolism. Compared with germ-free mice, specific pathogen-free mice showed increased alveolar bone loss and TRAP^+^ cells on the alveolar bone surface^25^. Another oral commensal *Streptococcus gordonii* induced bone resorption in vivo, induced osteoclast differentiation and inhibited osteoblast differentiation in vitro^26^. *S. gordonii* and its lipoproteins induced TNF-α and IL-1β through TLR2 in macrophages^27^ and IL-8 in periodontal ligament cells^28^. Oral symbiotic bacteria, especially viridians group streptococci (VGS), including *S. oralis* and *S. gordonii*, are commensal in human mucosa, but they are also regarded as opportunistic pathogens that can cause severe systemic diseases such as endocarditis and septic shock syndrome^29^. Even in periodontal disease or a healthy state, oral commensal bacteria always release EVs that can affect host immunity both locally and systemically. Since *S. oralis* EVs have potent TLR2-activating ability, VGS-derived EVs might be involved in systemic inflammation and progression of infectious diseases. Further studies are needed to identify the role of VGS-derived EVs in systemic diseases.

In addition to EVs derived from oral bacteria, we evaluated the effect of EVs derived from the probiotic strain *L. reuteri* on osteoclast differentiation. Lr EVs did not induce TLR activation, proinflammatory cytokine expression, or osteoclast differentiation. Probiotic-derived EVs have beneficial and anti-inflammatory effects in host environments^14^. Lr EVs inhibited LPS-induced proinflammatory responses in macrophages and splenic lymphocytes and showed protective effects against LPS-induced intestinal injury in chickens^30^. *Lactobacillus paracasei* EVs reduced LPS-induced proinflammatory responses in vitro and alleviated dextran sulfate sodium (DSS)-induced colitis in mice^31^. EVs from *Lactobacillus plantarum* increase M2 macrophage polarization and the expression of the anti-inflammatory cytokine IL-10 in human skin organ culture^32^. Although Lr EVs harbour TLR2 ligands such as lipoproteins, they did not induce TLR2 activation and osteoclastogenesis in our experimental settings. The inability of *L. reuteri* EVs to activate TLR2 could be explained by lipoprotein properties or the presence of immunomodulatory mediators. Lipoproteins of *L. reuteri* may have less immunogenic structures and lyso-form lipoproteins. *Lactobacillus bulgaricus* produces lyso-form lipoproteins^33^ that are produced by lipoprotein intramolecular transacylase (Lit)^34^. *L. reuteri* has a protein (Accession: WP_086118074.1) that has sequence similarity with the Lit of *L. bulgaricus* (Accession: MBT8800808.1). Lr EVs harbour diverse immunomodulatory mediators, including 60-kDa chaperonin (groEL), which efficiently increases anti-inflammatory cytokines IL-10 and TGF-β but does not induce the proinflammatory cytokines TNF-α and IL-12 in macrophages and dendritic cells^30,35^. The beneficial roles and therapeutic applications of Lr EVs on systemic bone diseases need to be further elucidated.

In the present study, we demonstrated that EVs derived from periodontal pathogens and oral commensal bacteria had osteoclastogenic activity via TLR2 activation, whereas probiotic-derived EVs did not. Bacterial EVs contain not only TLR ligands but also enzymes, toxins, and nucleic acids that can affect disease progression. Since periodontitis is closely related to systemic diseases and oral bacteria-derived EVs can migrate freely from local to systemic sites, further studies are needed to elucidate the role of pathogenic or non-pathogenic oral bacteria-derived EVs in periodontitis-related systemic diseases.

## Methods

### Reagents and chemicals

Brain heart infusion (BHI), Columbia broth, Lactobacilli MRS broth, yeast extract and Bacto agar were purchased from BD Biosciences (San Jose, CA, USA). l-Cysteine, l-arginine, resazurin, hemin, vitamin K, and *N*-acetylmuramic acid were purchased from Sigma (St. Louis, MO, USA). Alpha minimum essential medium (α-MEM) and phosphate-buffered saline (PBS) were purchased from Welgene (Daegu, South Korea). Penicillin/streptomycin (P/S), fetal bovine serum (FBS), and Ham’s F-12 medium were purchased from Gibco BRL (Paisley, UK). Recombinant murine soluble receptor activator of nuclear factor-κB ligand (RANKL) and macrophage colony-stimulating factor (M-CSF) were purchased from PeproTech (Rocky Hill, NJ, USA). G418, hygromycin B, Pam2CSK4, *Escherichia coli* LPS, ultrapure LPS, and ODN2006 were purchased from InvivoGen (San Diego, CA, USA). FITC anti-human CD25 mouse monoclonal antibody (clone#: M-A251) was purchased from BD Biosciences. Lipoprotein lipase from *Pseudomonas* species and polymyxin B were purchased from Sigma.

### Bacteria and extracellular vesicles

*P. gingivalis* (ATCC 33277), *T. forsythia* (ATCC 43037) and *F. alocis* (ATCC 35896) were cultured at 37°C in an anaerobic chamber (10% CO_2_, 10% H_2_, 80% N_2_). *Streptococcus oralis* (ATCC 9811) and *Lactobacillus reuteri* (ATCC 23272) were cultured at 37°C in an aerobic atmosphere. *P. gingivalis* was grown in BHI broth containing 5 μg/ml hemin and 1 μg/ml vitamin K. *T. forsythia* was grown in modified new oral spirochete broth (ATCC medium 1494 containing 0.25% yeast extract, 5 μg/ml hemin, 1 μg/ml vitamin K and 0.01 μg/ml *N*-acetylmuramic acid). *F. alocis* was grown in Columbia broth containing 5% yeast extract, 0.0025% resazurin, 5 μg/ml hemin, 1 μg/ml vitamin K, 1 μg/ml l-cysteine and 2 μg/ml l-arginine. *S. oralis* and *L. reuteri* were grown in BHI broth and Lactobacilli MRS broth, respectively. For EV isolation (1 L for one preparation), all bacteria were grown and harvested at 48 h or mid-log phase (12 h for *P. gingivalis*, 24 h for *T. forsythia*, 8 h for *S. oralis*, 16 h for *L. reuteri* and 24 h for *F. alocis*). At 48 h, the growth of *P. gingivalis*, *T. forsythia*, and *F. alocis* was in the late exponential phase, and the growth of *S. oralis* and *L. reuteri* was in the late stationary phase. After 48 h culture, the optical density (OD_600nm_) value was 1.0–1.2 (bacterial cell number, ≥ 1.1 × 10^13^ cells) for *P. gingivalis*, 0.8–1.0 (bacterial cell number, ≥ 8.2 × 10^12^ cells) for *T. forsythia*, 0.6–0.7 (bacterial cell number, ≥ 1.8 × 10^12^ cells) for *S. oralis*, 0.7–0.8 (bacterial cell number, ≥ 4.4 × 10^11^ cells) for *L. reuteri*, and 1.0–1.3 (bacterial cell number, ≥ 7.6 × 10^11^ cells) for *F. alocis*. OD_600nm_ and bacterial cell number were determined by spectrophotometer and heamocytometer, respectively.

Purification and characterization of bacterial EVs were performed as previously described^15^. Briefly, culture supernatants were filtered using a 0.22 μm polyethersulfone (PES) membrane filter (Corning, New York, NY, USA) and concentrated using a 100 kDa cut-off centricon plus-70 centrifugal filter (Merck, Darmstadt, Germany). The crude EVs were isolated by ultracentrifugation at 160,000×*g* at 4°C for 2 h using Optima XE-100 and Type 45 Ti rotor (Beckman Coulter, Brea, CA, USA). The EV containing pellet was resuspended with PBS and mixed with 60% iodixanol solution (Sigma) to make 40% crude EV/iodixanol solution. The 40% crude EV/iodixanol solution was laid on the bottom of the 14 ml ultracentrifuge tube, and overlaid with 35% and 10% of iodixanol solution, and subjected to buoyant density gradient ultracentrifugation (100,000×*g*, 4°C, 18 h) using an SW 40 Ti rotor (Beckman Coulter). Each fraction was obtained from top to bottom (fractions #1–10). The nanoparticle enriched fractions were determined by NTA using a NanoSight LM10 system and NTA 2.3 nanoparticle tracking and analysis software (Malvern Instruments Ltd, Worcestershire, UK). Fraction #4 and #5 were obtained and centrifuged at 160,000×*g* at 4°C for 2 h using SW40 Ti rotor. The pellets were dissolved in 500 µL PBS and stored at −80°C until use.

EV protein yield was measured by a bicinchoninic acid (BCA) assay kit (Thermo Fisher Scientific Inc., Waltham, MA, USA). The particle size and number of EVs were measured by NTA. The morphology of EVs were determined by TEM as previously described^36^. Briefly, bacterial EVs were plated on glow-discharged carbon-coated grids (Electron Microscopy Science) and negatively stained with 2% uranyl acetate. Bacterial EVs were observed using a LIBRA 120 electron microscope (Carl Zeiss).

### *Limulus amebocyte* lysate test

Endotoxin units of bacterial EVs were measured using a *Limulus amebocyte* lysate (LAL) chromogenic endotoxin quantification kit (Thermo Fisher Scientific Inc.) according to the manufacturer’s instructions.

### Osteoclast differentiation

All animal experiments were approved by the Institutional Animal Care and Use Committee (IACUC) of Seoul National University (SNU-210602-1-2) and conducted in accordance with the guidelines and regulation of the institute. The animal experiments were conducted in accordance with the ARRIVE guidelines (https://arriveguidelines.org). C57BL6 mice (8 weeks old, male) were purchased from Orient Bio (Gyeonggi-do, Korea), and TLR2^−/−^ mice (C57BL/6) were purchased from The Jackson Laboratory (Bar Harbor, ME, USA). The osteoclastogenic effects of bacterial EVs were determined as previously described^15^. Mice were euthanatized by carbon dioxide, and bone marrow-derived cells were suspended in complete α-MEM (10% FBS and 1% P/S), and plated on 100-mm cell culture plates with M-CSF (30 ng/ml) for 3 days. Then, to obtain osteoclast precursors, the cells (2 × 10^4^/well) were plated on 48-well plates with M-CSF (30 ng/ml) for 1 day and cultured with RANKL (60 ng/ml) and M-CSF (30 ng/ml) for an additional 3 days. Osteoclast precursors were stimulated with the 10 ug/ml of each bacterial EVs (EVs from 48 h culture or EVs from mid-log phase) in the presence of M-CSF (30 ng/ml) for 2 days. Particle numbers in 10 ug EV proteins were 1.6 × 10^12^ for Pg EVs, 1.87 × 10^12^ for Tf EVs, 1.26 × 10^12^ for So EVs, 1.01 × 10^12^ for Lr EVs, and 1.23 × 10^12^ for Fa EVs. After the stimulation period, mature osteoclasts were stained for tartrate-resistant acid phosphatase (TRAP) using a TRAP staining kit (Sigma) according to the manufacturer’s instructions. TRAP^+^ cells with ≥ 3 nuclei were regarded as mature osteoclasts and counted from three independent samples. Representative images of TRAP^+^ multinucleated cells (mature osteoclasts) were obtained from three independent samples by digital inverted microscopy (DS-Ri2, Nikon, Tokyo, Japan).

### Cytokine array and enzyme-linked immunosorbent assay

Osteoclast precursors were stimulated with the indicated stimuli in the presence of M-CSF (30 ng/ml) for 24 h. After the stimulation period, the culture supernatants were centrifuged (10,000×*g*, 4°C, 10 min) to exclude residual cell debris, and the supernatants were used. The cytokine expression profiles were measured using a Proteome Profiler Mouse Cytokine Array Kit, Panel A (R&D, Minneapolis, MN, USA), according to the manufacturer’s instructions. Proinflammatory cytokines (TNF-α, IL-6 and IL-1β) were measured by R&D ELISA kits.

### Toll-like receptor activation assay

The TLR2- or TLR4-activating abilities of bacterial EVs were analysed using CHO/CD14/TLR2 and CHO/CD14/TLR4 cells, respectively (Dr. Douglas Golenbock, Boston Medical Center, Boston, MA, USA). The cells were maintained in complete Ham’s F-12 medium containing G418 (1 mg/ml) and hygromycin B (0.4 mg/ml). CHO/CD14/TLR2 and CHO/CD14/TLR4 cells (3 × 10^5^/well) were plated in 48-well culture plates for 20 h and stimulated with the indicated stimuli for an additional 24 h. After the stimulation period, the cells were stained with FITC-conjugated anti-human CD25 antibodies. CD25^+^ cells were measured by flow cytometry (FACSCalibur, BD Biosciences) and analysed using FlowJo software (TreeStar, San Carlos, CA, USA). The TLR9-activating ability of bacterial EVs was analysed using HEK-Blue TLR9 cells (InvivoGen) according to the manufacturer’s instructions. Briefly, the cells were incubated with the indicated stimuli for 24 h. Secreted embryonic alkaline phosphatase (SEAP) by TLR9 activation was measured by a spectrophotometer at 620 nm.

### Statistical analysis

All experiments were performed at least three times. The data are represented as the mean values ± standard deviations. One-way analysis of variance (ANOVA) was performed to determine statistical significance among multiple groups with one independent variable. Two-way ANOVA performed to determine statistical significance among multiple groups with two independent variables. A *p value* of < 0.05 was considered statistically significant.

## Supplementary Information

Supplementary Information.

## Data Availability

The datasets used and/or analysed during the current study available from the corresponding author on reasonable request.
