# Supplementary material for: Effects of extracellular vesicles derived from oral bacteria on osteoclast differentiation and activation
Source: Sci Rep. 2022 Aug 20;12:14239. doi: 10.1038/s41598-022-18412-4 (PMC9396627; doi:10.1038/s41598-022-18412-4)
Supplement: Supplementary file 1 — Supplementary Information. [file 41598_2022_18412_MOESM1_ESM.docx]

**Supplementary Figure**


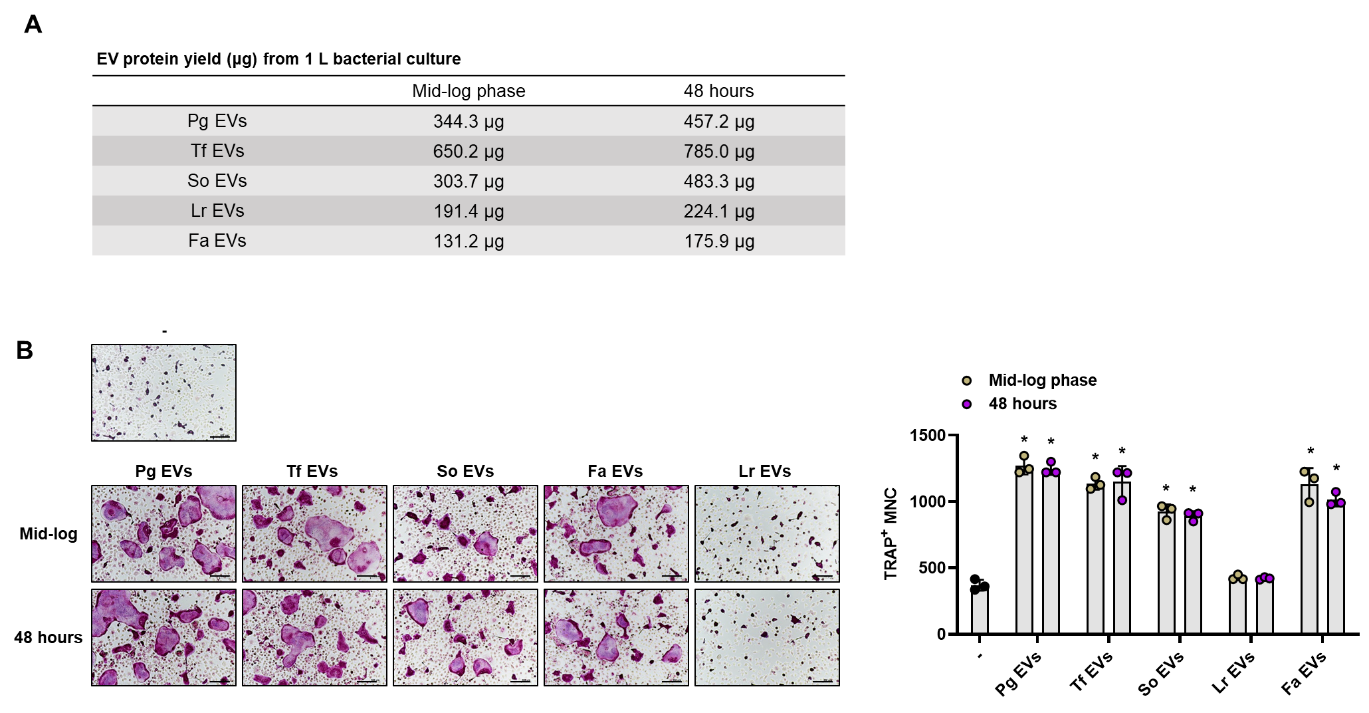


Supplementary Figure 1. Osteoclastogenic potencies of bacterial EVs isolated from mid-log phase and 48 hours. (A) Each EVs were isolated from mid-log phase (12 hours for *P. gingivalis*, 24 hours for *T. forsythia*, 8 hours for *S. oralis*, 16 hours for *L. reuteri* and 24 hours for *F. alocis*) and 48 hours bacterial culture. Protein concentration of EVs was measured by BCA assay. (B) Committed osteoclast precursors were stimulated with EVs (10 μg/ml EV protein) isolated from indicated growth phase in the presence of M-CSF (30 ng/ml) for 3 days. The mature osteoclasts were stained with TRAP, and representative images from triplicate samples are shown (*left panel*, scale bar: 200 μm). The number of TRAP^+^ mature osteoclasts was counted (*right* panel). The graphs show the mean values ± standard deviations of a representative experiment. Statistical significance was determined by two-way ANOVA. **P* < 0.05 compared with the nontreatment group (-).
